# Arabidopsis retromer subunit AtVPS29 is involved in SLY1-mediated gibberellin signaling

**DOI:** 10.1007/s00299-024-03144-8

**Published:** 2024-02-05

**Authors:** Wang Ki Min, Dae Hwan Kwon, Jong Tae Song, Hak Soo Seo

**Affiliations:** 1 Department of Agriculture, Forestry and Bioresources, College of Agriculture and Life Sciences, Seoul National University, Gwanakro 200, Gwanak-Gu, Seoul, 08826 Korea; 2 Department of Applied Biosciences, Kyungpook National University, Daegu, 41566 Korea

**Keywords:** Retromer protein, AtVPS29, Gibberellin, Meristematic zone, SLY1

## Abstract

**Key message:**

Retromer protein AtVPS29 upregulates the SLY1 protein and downregulates the RGA protein, positively stimulating the development of the root meristematic zone, which indicates an important role of AtVPS29 in gibberellin signaling.

**Abstract:**

In plants, the large retromer complex is known to play roles in multiple development processes, including cell polarity, programmed cell death, and root hair growth in Arabidopsis. However, many of its roles in plant development remain unknown. Here, we show that Arabidopsis trimeric retromer protein AtVPS29 (vacuolar protein sorting 29) modulates gibberellin signaling. The SLEEPY1 (SLY1) protein, known as a positive regulator of gibberellic acid (GA) signaling, exhibited lower abundance in *vps29-3* mutants compared to wild-type (WT) plants. Conversely, the DELLA repressor protein, targeted by the E3 ubiquitin ligase SCF (Skp, Cullin, F-box) complex and acting as a negative regulator of GA signaling, showed increased abundance in *vps29-3* mutants compared to WT. The *vps29-3* mutants exhibited decreased sensitivity to exogenous GA supply in contrast to WT, despite an upregulation in the expression of GA receptor genes within the *vps29-3* mutants. In addition, the expression of the GA synthesis genes was downregulated in *vps29-3* mutants, implying that the loss of AtVPS29 causes the downregulation of GA synthesis and signaling. Furthermore, *vps29-3* mutants exhibited a reduced meristematic zone accompanied by a decreased cell number. Together, these data indicate that AtVPS29 positively regulates SLY1-mediated GA signaling and plant growth.

## Introduction

Phytohormone GAs regulate plant development and growth at various developmental stages, including germination. For example, defects in GA synthesis or signaling cause non-germination or poor germination, severe dwarfism, and delayed flowering (Daviere and Achard 2013).

The stability and activity of proteins involved in signal transduction mediated by GA are regulated by post-translational modifications. First, the stability of DELLA proteins is regulated through the ubiquitination by the activity of single polypeptide or SCF complex E3 ubiquitin ligases. The F-box protein of the SCF complex plays a role in selecting target proteins for polyubiquitination by the E3 ligase activity of the SCF complex (Hua and Vierstra 2011). The Arabidopsis *SLY1* gene encodes the F-box subunit that acts as a bridge between Skp and DELLA proteins (McGinnis et al. 2003; Dill et al. 2004; Fu et al. 2004). DELLA proteins are transcriptional regulators that contain a conserved DELLA motif (Asp-Glu-Leu-Leu-Ala) in the N-terminal region and negatively regulate GA signaling. DELLA proteins directly interact with SLY1 and are rapidly degraded by the 26S proteasome complex after polyubiquitination. Proteins involved in GA signaling are also regulated by sumoylation pathway. SLY1 directly interacts with and is sumoylated by the E3 SUMO (Small Ubiquitin-related Modifier) ligase AtSIZ1 (Kim et al. 2015). The level of SLY1 is much lower in *siz1-2* mutants than in wild-type plants, and the level of sumoylated SLY1 is greatly increased by exogenous GA supply. DELLA protein RGA (REPRESSOR of *ga1-3*) is also sumoylated, although its E3 SUMO ligase has not been identified yet (Conti et al. 2014). Furthermore, RGA is more highly accumulated in the SUMO protease *ots1*/*ots2* mutants than in wild-type Arabidopsis, indicating that the SUMO-mediated pathway affects DELLA stability.

Retromers were initially characterized in yeast (Paravicini et al. 1992; Nothwehr and Hindes 1997; Seaman et al. 1997). They are protein complexes composed of a large subunit consisting of VPS26, VPS29, and VPS35 proteins and a small subunit consisting of sorting nexin dimers (Chandra et al. 2020). In yeast, this small subunit includes VPS5, while in animals and plants, it comprises SNX1/2 (Sorting Nexin 1/2) (Chandra et al. 2020). Retromers are responsible for transporting membrane proteins from the late endosome to the trans-Golgi network (Horazdovsky et al. 1997; Seaman et al. 1997; Tu and Seaman 2021). In plants, VPS26, VPS29, and VPS35 are localized to the prevacuolar compartment (Hashiguchi et al. 2010; Jha and Larson 2023) and exhibit potential interactions with each other (Jaillais et al. 2007; Zelazny et al. 2013; Jha and Larson 2023). These complexes are known to be involved in various plant developmental processes, including programmed cell death (Münch et al. 2015), oil body biogenesis and degradation during vegetative growth (Thazar-Poulot et al. 2015), and root hair growth (Jha et al. 2018; Jha and Larson 2023). Notably, VPS29 is implicated in the maturation of storage proteins 12S globulin and 2S albumin during seed development (Shimada et al. 2006), as well as in establishing cell polarity during plant organogenesis by influencing the subcellular trafficking of PIN proteins (Jaillais et al. 2007).

Accumulated data prove that SLY1 positively regulates growth through GA signaling. For instance, *sly1* mutants show increased seed dormancy and hypersensitivity to the inhibition of seed germination by abscisic acid (Strader et al. 2004). In addition, *sly1* plants display severe dwarfism and recover their phenotypes to the wild-type level in the absence of DELLA repressors (Dill et al. 2004; Fu et al. 2004; Olszewski et al. 2002). The *vps29-3* mutant also exhibits a severe dwarf phenotype commonly found in GA signaling-defective plants (Jaillais et al. 2007; Jha and Larson 2023). Notably, it has been reported that GA targets the retromer complex for trafficking modulation (Salanenka et al. 2018). Low GA levels promote the vacuolar delivery and degradation of multiple cargos, including PIN proteins, while high GA levels promote their recycling to the plasma membrane (PM). All of these data imply that SLY1-mediated GA signaling can be connected with protein trafficking by retromer complex.

Therefore, we questioned whether AtVPS29 affects GA signaling in Arabidopsis. We found that the level of SLY1 was much lower in the *vps29-3* mutant compared to the wild-type plants, while the DELLA protein RGA was highly accumulated in the *vps29-3* mutant. Both shoot and root growth of *vps29-3* mutants displayed reduced sensitivity to exogenous GA. In addition, *vps29-3* mutants showed a shortened meristematic zone and a decreased cell number. Taken together, we present evidence that AtVPS29 plays a significant regulatory role in plant development through its involvement in GA signaling.

## Materials and methods

### Plant materials and growth conditions

The *Arabidopsis thaliana* Columbia-0 ecotype (wild-type) and the T-DNA insertion knock-out mutant *vps29-3* were used in this study. The *vps29-3 *(Columbia accession, SALK010106) T-DNA mutant line was obtained from ABRC. For plants grown on plates, seeds were surface sterilized in commercial bleach containing 5% sodium hypochlorite and 0.1% Triton X-100 solution for 10 min, rinsed five times in sterilized water, and stratified at 4 °C for 2 days in the dark. Seeds were sown on agar plates containing Murashige and Skoog (MS) medium, 2% sucrose, and 0.8% agar, buffered to pH 5.7. For plants grown in soil, seeds were directly sown in sterile vermiculite. All plants, including seedlings, were grown at 22 °C under a 16 h light/8 h dark cycle in a growth chamber.

### Production and purification of the anti-AtVPS29 antibody

AtVPS29-specific antiserum was generated at Abcam Inc. Briefly, His_6_-tagged C-terminal AtVPS29 (amino acids, 172–190) was expressed in *E. coli* BL21 (DE3) and purified by affinity chromatography using Ni^2+^-nitrilotriacetate (Ni^2+^-NTA) resins (Qiagen). Antiserum was obtained from the rabbit immunized with purified His_6_-AtVPS29 peptides. Anti-AtVPS29 immunoglobulins were affinity purified from the antiserum by absorption with AtVPS29 protein bound to PVDF membrane.

### Examination of SLY1 and RGA levels in *vps29-3* mutants

To examine relative levels of SLY1 and RGA proteins in WT and *vps29-3* mutants, total proteins were extracted from the leaves of WT and *vps29-3* mutants grown for 15 d on MS media. Following 11% SDS-PAGE, the levels of SLY1 and RGA proteins were assessed by western blotting using anti-SLY1 or anti-RGA antibodies. Tubulin levels were also examined by western blotting with anti-tubulin antibody (Santa Cruz Biotechnology). Western blotting was performed five times, and one of these results was presented.

### Transcript levels of genes related to GA signaling in *vps29-3* mutants

WT and *vps29-3* mutants were grown on plates containing MS medium for 15 d. Total RNA was extracted from the leaves of both WT and *vps29-3* mutants. The extracted RNA was then quantified and divided into equal amounts. First-strand cDNA was synthesized from 5 μg total RNA using an iScript cDNA Synthesis Kit (Bio-Rad). An equal volume of cDNA was amplified by real-time qRT-PCR (MyiQ, Bio-Rad), according to the manufacturer’s protocol. The specific primers and template cDNA were combined with 25 µl of iQ SYBR Green Super Mix (Bio-Rad), and the reactions were performed under the following thermal conditions: 50 °C for 2 min; 95 °C for 10 min; 40 cycles of 95 °C for 15 s and 60 °C for 1 min. The C_T_ values obtained for target genes were normalized to the C_T_ value for tubulin, and the data were analyzed using iCycler IQ software (Bio-Rad). PCR primers were designed using Primer3 (http://frodo.wi.mit.edu/cgi-bin/primer3/primer3.cgi), and their specificity was verified by cloning into the pGEM T-Easy vector (Promega) and sequencing using an ABI 3730xl DNA Analyzer (Applied Biosystems). The primers used for qRT-PCR were as follows: *SLY1*, 5′-TGAAACACGTCGACGCAAAG-3′ and 5′-TCGTCTTGTGCCGTTTTGTG-3′; *RGA*, 5′-AACCAAGCGATTCTCGAAGC-3′ and 5′-AAGTGCAGGCCATTGAAGAC-3′; *GID1a*, 5′-TGGGGTTTTCTCGTTCGATG-3′ and 5′-ACAATGTCGCCATCAACAGG-3′; *GID1b*, 5′-5′-TGCGGTGTTGTTGTTGTCTC-3′ and 5′-ATCGTCGTAAGCACAAGGGTAG-3′; *GID1c*, 5′-TTGGAGGGACCGAAAGAACG-3′ and 5′-TCACCCTCAGGAAGAAACGC-3′; *GA20ox2*, 5′-GCTCAGAGAAAACCCGGTGA-3′ and 5′-TCTTGAACGGTTCTCGAGCC-3′; *GA3ox1*, 5′-GATCTCCTCTTCTCCGCTGC-3′ and 5′-TTTGGAAGGCACCCCAAGTT-3′; *GA3ox2*, 5′-TAGATCGCATCCCATTCACA-3′ and 5′-TCAATGTCGTCGAGAAGTCG-3′; *Tubulin*, 5′-GTGAGCGAACAGTTCACAGC-3′ and 5′-TTATTGCTCCTCCTGCACTT -3′.

**Shoot and root growth analysis of vps29-3 mutants:** WT and *vps29-3* mutants were germinated on 1/2 MS agar-solidified medium (1% agar for vertical growth) supplemented with 0, 10, and 20 µM GA_3_, respectively. After growth for 8 days on vertical plates, shoot sizes and root lengths were measured using the ImageJ software with scanned images of seedlings (Schindelin et al. 2012).

### Analysis of root apical meristem size of *vps29-3* mutants

Arabidopsis seedlings were germinated in 1/2 MS agar-solidified medium (1% agar for vertical growth) supplemented with 1% sucrose. To quantify the number of the cortex cells and measure cell lengths at the meristematic region, roots were stained with 10 μg/ml propidium iodide (PI, Sigma-Aldrich) for 2 min. Confocal images were obtained using a Leica TCS SP8 confocal microscope equipped with a 561-nm laser. The numbers and lengths of cortical cells between the quiescent center and the first elongated cell were measured using the ImageJ software (Schindelin et al. 2012).

## Results

### The level of SLY1 is influenced by retromer protein AtVPS29

**Fig. 1:**
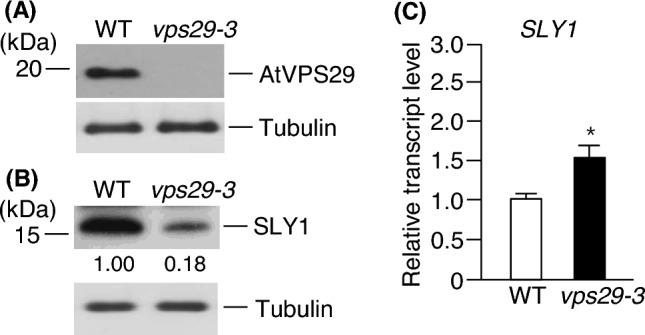
AtVPS29 positively regulates SLY1 level but negatively regulates its expression. Total proteins were extracted from leaves of 15-day-old wild-type (WT) and *vps29-3* mutants. After performing 11% SDS-PAGE, the levels of AtVPS29 and SLY1 were examined using western blotting with anti-AtVPS29 (**a**) or anti-SLY1 (**b**) antibodies. Numbers under the lanes indicate relative intensities. Tubulin was used as a loading control. **c** Total RNA was isolated from 15-day-old WT and *vps29-3* mutant plants. Transcript levels were assessed using real-time qRT-PCR with *SLY1*-specific primers. Results are presented as means ± S.D. (*n* = 3). *Asterisks* indicate statistically significant differences in transcript levels (^*^p < 0.05; Student’s *t *test) between WT and *vps29-3* mutants. SLY1, SLEEPY1

Abnormal GA signaling caused by the loss of SLY1 function results in defective growth, leading to severe dwarfism and low fertility (McGinnis et al. 2003). Arabidopsis *vps29-3* mutants have also been reported to exhibit severe dwarfism and abnormal seed development (Shimada et al. 2006; Jaillais et al. 2007). Furthermore, the regulation of PM protein accumulation by GA requires the presence of retromer complex components (Salanenka et al. 2018). These suggest that SLY1 activity may be changed in the *vps29-3* mutant, leading to abnormal SLY1-mediated GA signaling in the *vps29-3* mutant. To test this hypothesis, we first generated an anti-AtVPS29 antibody and examined its specificity through western blotting. We observed clear detection of the AtVPS29 protein in WT, while it was not detected in *vps29-3* plants, indicating that the antibody specifically interacts with VPS29 (Fig. 1a). Based on this result, we proceeded to analyze the levels of SLY1 protein in WT and *vps29-3* mutants using western blotting with an anti-SLY1 antibody. Interestingly, we found that the level of SLY1 protein was significantly lower in the *vps29-3* mutants (Fig. 1b). The SLY1 concentration in *vps29-3* mutants was approximately 5.56-fold lower than that in WT. To investigate whether the reduced level of SLY1 protein in the *vps29-3* mutants is influenced by the transcript level of *SLY1*, we performed real-time qRT-PCR analysis of *SLY1* transcripts in the same samples. Surprisingly, the results showed that the transcript level of SLY1 was actually higher in the *vps29-3* mutants compared to WT (Fig. 1c).

### Expression of GA receptor and biosynthesis genes was changed in *vps29-3* mutants

**Fig. 2:**
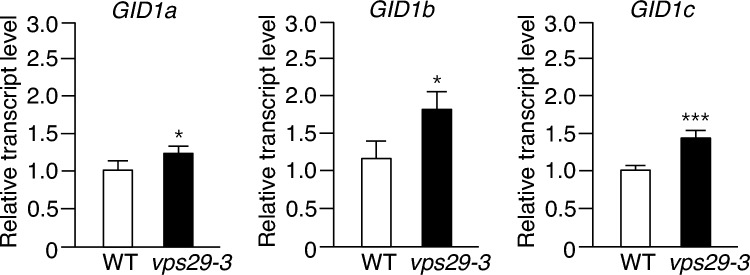
Transcript levels of GA receptor genes in *vps29-3* mutants. Total RNA was isolated from 15-day-old WT and *vps29-3* mutants. Transcript levels were analyzed using real-time qRT-PCR with gene-specific primers. Results are presented as means ± S.D. (*n* = 3). *Asterisks* indicate statistically significant differences in transcript levels (^*^p < 0.05; ^***^p < 0.001; Student’s *t *test) between WT and *vps29-3* mutants. Abbreviations: *GID1a*, *GA-INSENSITIVE DWARF 1a*; *GID1b*, *GA-INSENSITIVE DWARF 1b*; *GID1c*, *GA-INSENSITIVE DWARF 1c*

We have demonstrated that the level of SLY1 protein is low in *vps29-3* mutants, suggesting that the defective growth observed in these mutants may be partially attributed to the reduced level and activity of SLY1. However, it is also plausible that the dwarfism observed in *vps29-3* mutants is a consequence of altered gene expression. Therefore, we investigated the transcript levels of the GA receptor genes *GA-INSENSITIVE DWARF 1a (GID1a)*, *GID1b*, and *GID1c* in *vps29-3* mutants. To assess the expression of these genes, we performed real-time qRT-PCR using total RNA isolated from both WT and *vps29-3* mutants. Notably, our results revealed that the expression of *GID1a*, *GID1b*, and *GID1c* genes was significantly increased in *vps29-3* mutants compared to WT (Fig. 2).

**Fig. 3:**
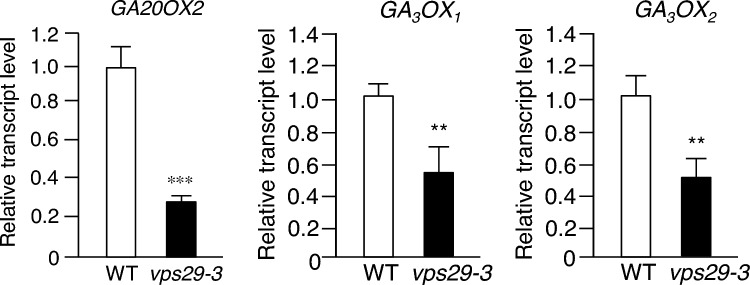
Transcript levels of GA-synthetic genes in *vps29-3* mutants. Total RNA was isolated from 15-day-old WT and *vps29-3* mutants. Transcript levels were analyzed using real-time qRT-PCR with gene-specific primers. Results are presented as means ± S.D. (*n* = 3). *Asterisks* indicate statistically significant differences in transcript levels (^**^p < 0.01; ^***^p < 0.001; Student’s *t *test) between WT and *vps29-3* mutants. Abbreviations: *GA20ox2*, *GIBBERELLIN 20-OXIDASE 2*; *GA3ox1*, *GIBBERELLIN 3-OXIDASE 1*; *GA3ox2*, *GIBBERELLIN 3-OXIDASE 2*

Next, we investigated the transcript levels of GA biosynthetic genes, namely *GA20ox2*, *GA3ox1*, and *GA3ox2*, under the same conditions in *vps29-3* mutants. Interestingly, we observed a significant decrease in the levels of *GA20ox2*, *GA3ox1*, and *GA3ox2* transcripts in *vps29-3* mutants compared to WT (Fig. 3).

### The level of RGA protein is affected by AtVPS29

**Fig. 4:**
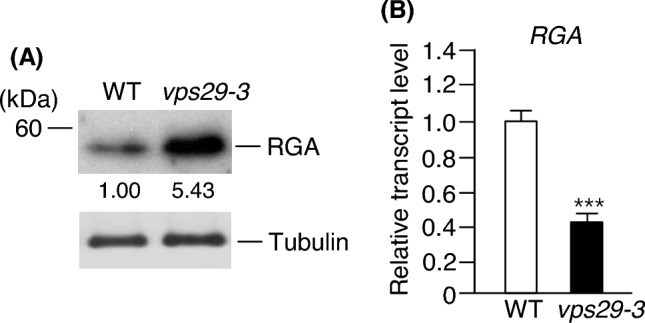
AtVPS29 negatively regulates RGA level but positively regulates its expression. **a** Total proteins were extracted from leaves of 15-day-old WT and *vps29-3* mutants. After subjecting them to 11% SDS-PAGE, the levels of RGA were analyzed by western blotting using anti-RGA antibody. Numbers under the lanes indicate relative intensities. Tubulin was used as a loading control. **b** Total RNA was isolated from 15-day-old WT and *vps29-3* mutants. Transcript levels were determined using real-time qRT-PCR with *RGA*-specific primers. Results are expressed as means ± S.D. (*n* = 3). *Asterisks* indicate statistically significant differences in transcript levels (^***^p < 0.001; Student’s *t *test) between WT and *vps29-3* mutants. *RGA*, *REPRESSOR OF ga1-3*

In Arabidopsis, the F-box protein SLY1 plays a crucial role in recognizing and targeting DELLA proteins, including RGA, for degradation by the 26S proteasome complex. Given the decreased level of SLY1 protein in *vps29-3* mutants (Fig. 1b), we initially investigated the levels of RGA in both WT and *vps29-3* mutants using western blotting with anti-RGA antibody. The results demonstrated a significant accumulation of RGA protein in the *vps29-3* mutants compared to WT (Fig. 4a). The RGA concentration in *vps29-3* mutants was approximately up to 5.43-fold higher than that in WT. Next, we examined the impact of *RGA* gene expression on the level of RGA protein. Transcript levels of the *RGA* gene were determined by real-time qRT-PCR using total RNA isolated from the same samples. Interestingly, we observed a lower transcript level of *RGA* in the *vps29-3* mutants compared to WT (Fig. 4b).

### *vps29-3* mutants are less sensitive to gibberellin

**Fig. 5:**
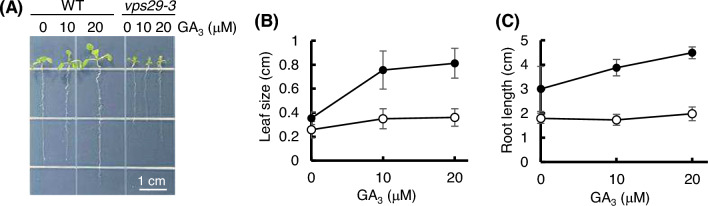
*vps29-3* mutants are less sensitive to exogenous GA. **a** Comparison of leaf size and root length between WT and *vps29-3* mutants after 8 days of growth on 1/2 MS medium (mock) and 1/2 MS medium supplemented with 10 and 20 µM GA_3_, respectively. *Scale bar* 1 cm. **b**, **c** Statistical analysis of the differences in leaf size (**b**) and root length (**c**) between the genotypes shown in (**a**). Data are presented as means ± S.D (*n* > 10). Black and white circles indicate WT and *vps29-3* mutants, respectively

To assess the GA response of *vps29-3* mutants, we germinated seeds from both WT and *vps29-3* mutants on MS medium supplemented with 10 and 20 µM GA_3_. After 8 days, we measured leaf size and root length. When exposed to increasing concentrations of GA_3_ ranging from 10 to 20 μM, leaf growth and root elongation in WT exhibited a dose-dependent stimulation (Fig. 5a–c). In contrast, GA_3_ had minimal effects on leaf growth and root elongation in *vps29-3* mutants. Specifically, leaf size and root length showed a 2.56-fold and 1.50-fold increase, respectively, in WT. Conversely, leaf size and root length exhibited only a 1.40-fold and 1.10-fold increase, respectively, in *vps29-3* mutants.

### The length of root meristematic zone was reduced in *vps29-3* mutants

**Fig. 6:**
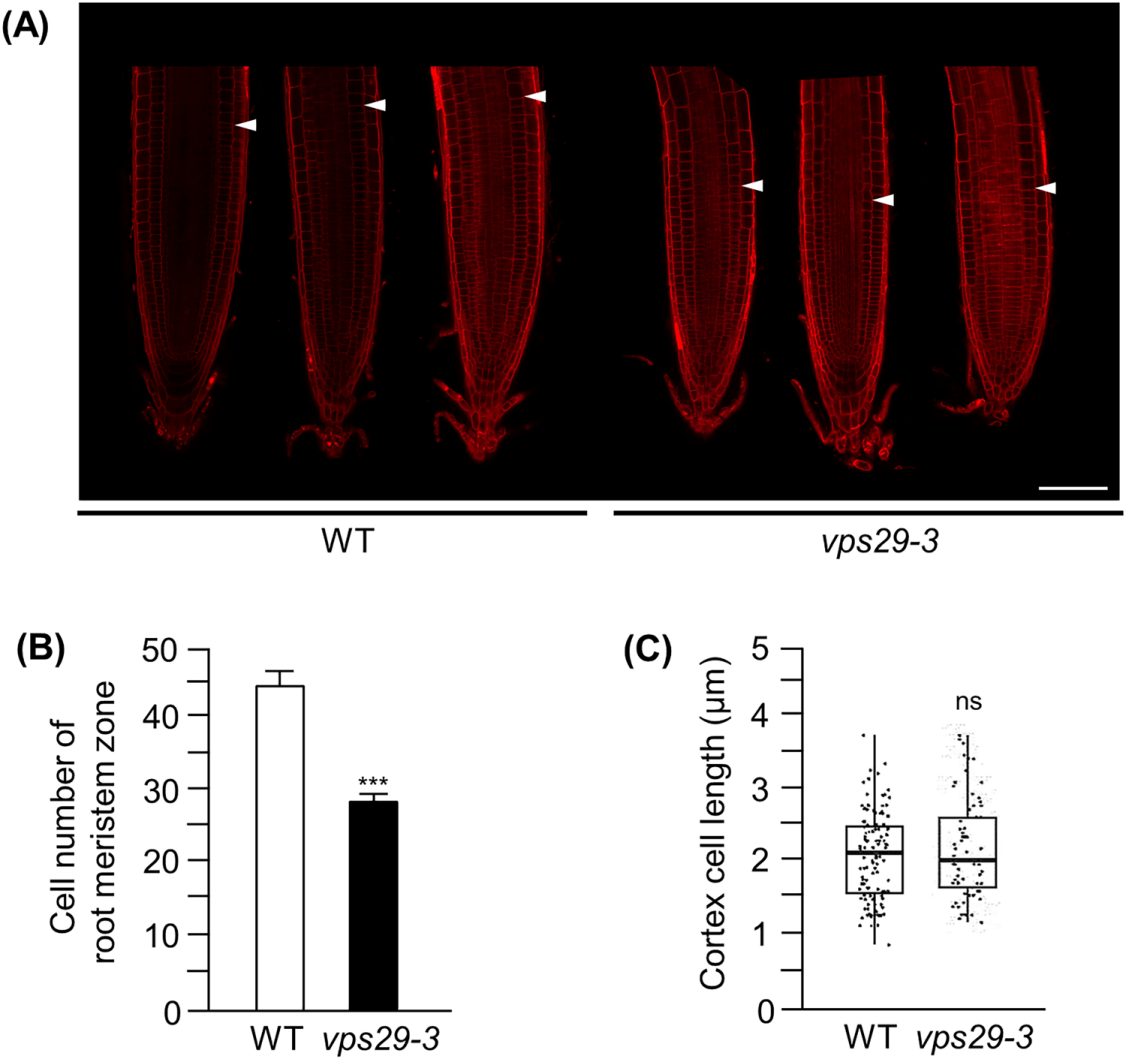
*vps29-3* mutants show reduced root meristem size. **a** Confocal images of root tips of WT and *vps29-3* mutants. Roots from seedlings 7 days after germination were used for imaging. White arrowheads indicate the boundary between the meristematic and elongation zones of the root. *Scale bars* correspond to 25 μm. **b** Root meristem cell number in the roots of 7-day-old WT and *vps29-3* mutants. *Asterisks* indicate statistically significant differences in transcript levels (^***^p < 0.001; Student’s *t* test) between WT and *vps29-3* mutants. Values represent the mean of 15 measurements ± SD. **c** Average cortex cell sizes in the roots of 6-day-old WT and *vps29-3* mutants. Each value represents measurements from at least 15 seedlings. The median, minimum, and maximum values are shown. NS, not significant

GA is an important regulator of *Arabidopsis* root growth (Fu and Harberd 2003; Griffiths et al. 2006; Willige et al. 2007; Ueguchi-Tanaka et al. 2007; Ubeda-Tomás et al. 2008). Reduction of endogenous GA levels by treating WT seedlings with paclobutrazol (PAC, an inhibitor of GA biosynthesis) results in a reduced root growth rate and also in a reduction in root meristem size (Ubeda-Tomás et al. 2009). Therefore, the decrease in SLY1 protein and the increase in RGA protein in *vps29-3* mutants, along with reduced sensitivity to GA, strongly suggest that root growth is impaired in *vps29-3* mutants. Consequently, we examined the size of the root meristematic zone in 5-day-old WT and *vps29-3* mutants. Propidium iodide staining revealed that the length of the meristematic zone was much shorter in *vps29-3* mutants compared to WT (Fig. 6a). This observation implies that either cell number or length is affected in *vps29-3* mutants. Further analyses indicated that cell number was decreased in *vps29-3* mutants (Fig. 6b), while cortex cell size remained unaffected (Fig. 6c).

## Discussion

This study emphasizes the positive regulatory role of retromer protein AtVPS29 activity in plant development through SLY1-mediated GA signaling.

GA-dependent developmental processes are influenced by DELLA proteins, which act as repressors and are regulated through phosphorylation and ubiquitination mediated by the E3 ligase activity of SCF^SLY1^. Previous studies have extensively investigated SLY1 and DELLA proteins to unravel the mechanisms of GA signaling. Notably, SLY1- and DELLA-mediated growth are also modulated by sumoylation.

The severe dark green dwarfism observed in Arabidopsis *sly1* mutants is a prominent characteristic. Interestingly, a similar dwarf phenotype is observed in *vps29-3* mutants. Hence, we aimed to investigate whether a VPS29 mutation affects GA signaling by disrupting SLY1-mediated degradation of DELLA proteins. Our results revealed lower levels of SLY1 protein in *vps29-3* mutants compared to WT (Fig. 1b), despite higher transcript levels in *vps29-3* mutants (Fig. 1c). This strongly suggests that AtVPS29 activity plays a role in stabilizing SLY1 protein post-translationally. GA signaling is primarily regulated by the negative activity of DELLA proteins, which undergo degradation by the 26S proteasome complex following polyubiquitination by SCF^SLY1^ (Dill et al. 2004). Our findings demonstrated that the level of SLY1 protein was positively regulated by AtVPS29 activity (Fig. 1b), implying that the abundance of DELLA proteins, including RGA, might be influenced in *vps29-3* mutants. Remarkably, the level of RGA was significantly elevated in *vps29-3* mutants compared to WT (Fig. 4a), while its transcript level was decreased in *vps29-3* mutants (Fig. 4b). We found that the expression patterns of the *SLY1* and *RGA* genes show no correlation with their protein levels. This implies that AtVPS29 loss might influence various signaling pathways, impacting not only the expression of SLY1 and RGA but also the post-translational modification systems. Several studies have indicated a lack of clear correlation between transcript and protein levels. Richter et al. (2010) demonstrated an absence of correlation between DELLA protein abundance and *DELLA* gene expression during late germination stages and following GA_3_ treatment. This supports the notion that feedback mechanisms likely regulate DELLA protein levels through a balance of synthesis and degradation. Li et al. (2015) observed reduced transcription levels of *RGA* genes in *det1-1* mutants compared to the wild-type under darkness. Interestingly, the mutants exhibited higher abundance of the RGA protein, suggesting a potential post-translational role of De-etiolated 1 (DET1) in elevating RGA protein level during dark condition. Zia et al. (2019) found that although the expression of the *IAA28* gene was induced by auxin treatment, its protein level decreased. This suggests a discordance between gene expression and protein abundance in response to auxin. Therefore, based on these findings, we cautiously speculate that similar mechanisms might regulate the expression of *SLY1* and *RGA* genes, as well as the degradation of their respective proteins in *vps29-3* mutants, although the exact mechanisms remain unclear. Moreover, all these results support the notion that AtVPS29 stimulates SLY1-mediated degradation of RGA. Therefore, these findings strongly indicate that the dwarfism observed in *vps29-3* mutants is attributable to the loss of SLY1 activity.

However, it is also plausible that the growth defects in *vps29-3* mutants may arise from alterations in the expression of genes involved in GA synthesis and response. The data revealed decreased transcript levels of genes encoding GA biosynthetic enzymes in *vps29-3* mutants (Fig. 3), suggesting that the loss of AtVPS29 leads to the downregulation of GA synthesis. However, transcript levels of genes encoding GA receptors were increased in *vps29-3* mutants (Fig. 2). Previous studies have demonstrated that *GA20ox2*, *GA3ox1*, *GID1a*, and *GID1b*, responsible for encoding GA biosynthetic enzymes and GA receptors, are downregulated by GA treatment and loss-of-function DELLA mutations (Hedden and Phillips 2000; Fleets and Sun 2005; Griffiths et al. 2006; Zentella et al. 2007), implying their expression is positively regulated by DELLA proteins. However, Zentella et al. (2007) also reported an intriguing finding: in a DEX-inducible transgenic system, they observed an increase in the transcript levels of *GA3ox1*, *GA20ox2, GID1a*, and *GID1b* genes upon *RGA* induction. This suggests that DELLA proteins not only act as repressors of GA signaling but also modulate GA homeostasis by potentially upregulating the expression of GA biosynthetic and GA receptor genes. Based on these findings, we cautiously speculate that the expression of downstream genes of *RGA* in the *vps29-3* mutants could be regulated not only by a negative feedback loop through DELLA proteins but also by another potential alternative mechanism. In addition, despite the increased expression of genes encoding GA receptors in *vps29-3* mutants, the responsiveness of *vps29-3* mutants to GA in terms of shoot and root growth was substantially diminished compared to WT (Fig. 5a–c), suggesting reduced sensitivity to GA in *vps29-3* mutants.

Plant growth involves the integration of various environmental and endogenous signals that, in conjunction with the intrinsic genetic program, determine plant size. At the cellular level, growth rate is governed by two interconnected processes: cell proliferation and expansion. GA plays a pivotal role in regulating growth and development in response to environmental fluctuations (Olszewski et al. 2002; Achard et al. 2006, 2008). Given the impairment of the SLY1-mediated signaling pathway (Figs. 1b and 4a), reduced GA response (Fig. 5a–c), and shortened root length in *vps29-3* mutants (Fig. 5a), we, therefore, expanded our investigation to include the measurement of the root meristematic zone length and cell number and length within this zone. The results unequivocally demonstrated that *vps29-3* mutants had a shortened meristematic zone (Fig. 6a), accompanied by a decline in cell count within the meristematic zone (Fig. 6b). This implies that the decrease in cell number within the meristematic zone of *vps29-3* mutants resulted from impaired GA signaling, ultimately contributing to a reduction in the length of the meristematic zone.

**Fig. 7:**
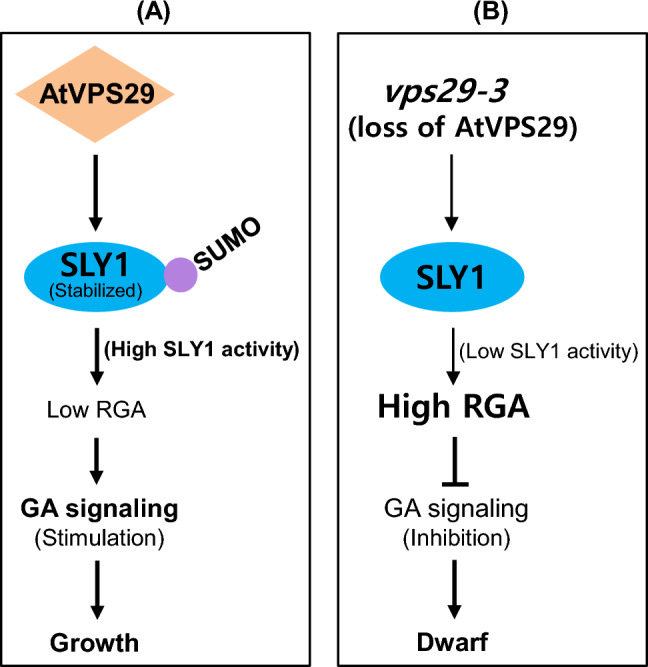
Schematic representation of the possible mechanism of activation of GA signaling by AtVPS29. **a** In wild-type plants, SLY1 undergoes sumoylation via AtSIZ1 activity, which converts it into its active form. Sumoylated SLY1 forms the SCF^SLY1−SUMO^ complex, which targets DELLA proteins, including RGA, for ubiquitination. Polyubiquitinated DELLA proteins are subsequently degraded by the 26S proteasome complex. This process promotes cell division and expansion, leading to growth and development. **b** Due to potentially reduced AtSIZ1 activity in *vps29-3* mutants, SLY1 might not undergo sumoylation, leading to its inactivation and consequent accumulation of RGA. As a result, cell division and expansion are inhibited, leading to dwarfism

SLY1 undergoes direct sumoylation by AtSIZ1 both in vitro and in vivo, and the level of SLY1 protein is reduced in *siz1-2* mutants compared to WT (Kim et al. 2015). In addition, GA promotes the sumoylation of SLY1 by AtSIZ1 (Kim et al. 2015). Moreover, Arabidopsis *siz1-2* mutants exhibit a severe dwarf phenotype (Catala et al. 2007) because the accumulation of DELLA repressors in the absence of SLY1 inhibits cell elongation and division (Achard et al. 2009; Peng et al. 1997; Dill et al. 2001). These data indicate that AtSIZ1 stabilizes SLY1 through its E3 ligase activity, positively regulating SLY1-mediated GA signaling and thereby influencing plant growth. In the present study, we observed a notable reduction in the level of SLY1 in *vps29-3* mutants compared to WT (Fig. 1b). Furthermore, the level of RGA was considerably elevated in *vps29-3* mutants in contrast to WT (Fig. 4a), implying a potential decrease in AtSIZ1 levels or activity in *vps29-3* mutants. Therefore, these findings, together with previous our data, suggest the possibility that impaired SLY1-mediated GA signaling in *vps29-3* mutants could be due to reduced SLY1 activity resulting from diminished AtSIZ1 activity (Fig. 7). Recently, several proteins associated with the regulation of retromer protein function have been identified in plants. These include the endosomal sorting complex required for transport (ESCRT)-associated protein apoptosis-linked gene-2 interacting protein X (ALIX) (Hu et al. 2022), BLISTER (Li et al. 2023), MoRab7, and the DEAH and RING domain-containing protein as FREE1 suppressor 1 (DRIF1) (Chen et al. 2023; Zhu et al. 2023). They play a role in regulating protein transport and signaling by interacting with retromer proteins. These findings suggest the involvement of various proteins in the regulation of the retromer complex’s function in plants.

In conclusion, AtVPS29 exerts positive and negative regulatory effects on the levels of SLY1 and RGA proteins, respectively, primarily at the post-translation level. The depletion of AtVPS29 results in reduced responsiveness to exogenous GA, a shortened meristematic zone, and a decrease in cell numbers within meristematic zone. These findings provide strong evidence suggesting that AtVPS29, a component of the large retromer complex, plays a significant role in modulating GA signaling, consequently influencing plant growth.

## Data Availability

The data that support the findings of this study are available from the corresponding author upon reasonable request.

## References

[CR1] Achard P, Cheng H, De Grauwe L, Decat J, Schoutteten H, Moritz T, Van Der Straeten D, Peng J, Harberd NP (2006). Integration of plant responses to environmentally activated phytohormonal signals. Science.

[CR2] Achard P, Renou J-P, Berthomé R, Harberd NP, Genschik P (2008). Plant DELLAs restrain growth and promote survival of adversity by reducing the levels of reactive oxygen species. Curr Biol.

[CR3] Achard P, Gusti A, Cheminant S, Alioua M, Dhondt S, Coppens F, Beemster GT, Genschik P (2009). Gibberellin signaling controls cell proliferation rate in Arabidopsis. Curr Biol.

[CR4] Catala R, Ouyang J, Abreu IA, Hu Y, Seo H, Zhang X, Chua NH (2007). The Arabidopsis E3 SUMO ligase SIZ1 regulates plant growth and drought responses. Plant Cell.

[CR5] Chandra M, Kendall AK, Jackson LP (2020). Unveiling the cryo-EM structure of retromer. Biochem Soc Trans.

[CR6] Chen X, Selvaraj P, Lin L, Fang W, Wu C, Yang P, Zhang J, Abubakar YS, Yang F, Lu G, Liu W, Wang Z, Naqvi NI, Zheng W (2023). Rab7/Retromer-based endolysosomal trafficking is essential for proper host invasion in rice blast. New Phytol.

[CR7] Conti L, Nelis S, Zhang C, Woodcock A, Swarup R, Galbiati M, Tonelli C, Napier R, Hedden P, Bennett M, Sadanandom A (2014). Small Ubiquitin-like Modifier protein SUMO enables plants to control growth independently of the phytohormone gibberellin. Dev Cell.

[CR8] Daviere J, Achard P (2013) Gibberellin signaling in plants. Development 140:1147–1151 10.1242/dev.087650 23444347

[CR9] Dill A, Jung HS, Sun TP (2001). The DELLA motif is essential for gibberellin-induced degradation of RGA. Proc Natl Acad Sci USA.

[CR10] Dill A, Thomas SG, Hu J, Steber CM, Sun TP (2004). The Arabidopsis F-box protein SLEEPY1 targets gibberellin signaling repressors for gibberellin-induced degradation. Plant Cell.

[CR11] Fleet CM, Sun TP (2005). A DELLAcate balance: the role of gibberellin in plant morphogenesis. Curr Opin Plant Biol.

[CR12] Fu X, Harberd NP (2003). Auxin promotes Arabidopsis root growth by modulating gibberellin response. Nature.

[CR13] Fu X, Richards DE, Fleck B, Xie D, Burton N, Harberd NP (2004). The Arabidopsis mutant *sleepy1*^*gar2-1*^ protein promotes plant growth by increasing the affinity of the SCF^SLY1^ E3 ubiquitin ligase for DELLA protein substrates. Plant Cell.

[CR14] Griffiths J, Murase K, Rieu I, Zentella R, Zhang ZL, Powers SJ, Gong F, Phillips AL, Hedden P, Sun TP, Thomas SG (2006). Genetic characterization and functional analysis of the GID1 gibberellin receptors in Arabidopsis. Plant Cell.

[CR15] Hashiguchi Y, Niihama M, Takahashi T, Saito C, Nakano A, Tasaka M, Morita MT (2010). Loss-of-function mutations of retromer large subunit genes suppress the phenotype of an *Arabidopsis* zig mutant that lacks Qb-SNARE VTI11. Plant Cell.

[CR16] Hedden P, Phillips AL (2000). Gibberellin metabolism: New insights revealed by the genes. Trends Plant Sci.

[CR17] Horazdovsky BF, Davies BA, Seaman MN, McLaughlin SA, Yoon S, Emr SD (1997). A sorting nexin-1 homologue, Vps5p, forms a complex with Vps17p and is required for recycling the vacuolar protein-sorting receptor. Mol Biol Cell.

[CR18] Hu S, Li B, Wu F, Zhu D, Zouhar J, Gao C, Shimada T, Rojo E, Hara-Nishimura I, Jiang L, Shen J (2022). Plant ESCRT protein ALIX coordinates with retromer complex in regulating receptor-mediated sorting of soluble vacuolar proteins. Proc Natl Acad Sci USA.

[CR19] Hua Z, Vierstra RD (2011). The cullin-RING ubiquitin-protein ligases. Annu Rev Plant Biol.

[CR20] Jaillais Y, Santambrogio M, Rozier F, Fobis-Loisy I, Miège C, Gaude T (2007). The retromer protein VPS29 links cell polarity and organ initiation in plants. Cell.

[CR21] Jha SG, Larson ER (2023). Diversity of retromer-mediated vesicular trafficking pathways in plants. Front Plant Sci.

[CR22] Jha SG, Larson ER, Humble J, Domozych DS, Barrington DS, Tierney ML (2018). Vacuolar Protein Sorting 26C encodes an evolutionarily conserved large retromer subunit in eukaryotes that is important for root hair growth in *Arabidopsis thaliana*. Plant J.

[CR23] Kim SI, Park BS, Kim DY, Yeu SY, Song SI, Song JT, Seo HS (2015). E3 SUMO ligase AtSIZ1 positively regulates SLY1-mediated GA signalling and plant development. Biochem J.

[CR24] Li K, Gao Z, He H, Terzaghi W, Fan LM, Deng XW, Chen H (2015). *Arabidopsis* DET1 represses photomorphogenesis in part by negatively regulating DELLA protein abundance in darkness. Mol Plants.

[CR25] Li H, Huang R, Liao Y, Yang S, Feng B, Qin H, Zhou J, Zeng Y, Shen J, Zhuang X, Jiang L, Otegui MS, Zhang S, Gao C (2023) A plant-unique protein BLISTER coordinates with core retromer to modulate endosomal sorting of plasma membrane and vacuolar proteins. Proc Natl Acad Sci USA 120(1):e2211258120 10.1073/pnas.2211258120 PMC9910430 36577063

[CR26] McGinnis KM, Thomas SG, Soule JD, Strader LC, Zale JM, Sun TP, Steber CM (2003). The Arabidopsis *SLEEPY1* gene encodes a putative F-box subunit of an SCF E3 ubiquitin ligase. Plant Cell.

[CR27] Münch D, Teh OK, Malinovsky FG, Liu Q, Vetukuri RR, Kasmi FE, Brodersen P, Hara-Nishimura I, Dangle JL, Petersen M, Mundy J, Hofius D (2015). Retromer contributes to immunity-associated cell death in Arabidopsis. Plant Cell.

[CR28] Nothwehr SF, Hindes AE (1997). The yeast VPS5/GRD2 gene encodes a sorting nexin-1-like protein required for localizing membrane proteins to the late Golgi. J Cell Sci.

[CR29] Olszewski N, Sun TP, Gubler F (2002). Gibberellin signaling: biosynthesis, catabolism, and response pathways. Plant Cell.

[CR30] Paravicini G, Horazdovsky BF, Emr SD (1992). Alternative pathways for the sorting of soluble vacuolar proteins in yeast: a *vps35* null mutant missorts and secretes only a subset of vacuolar hydrolases. Mol Biol Cell.

[CR31] Peng J, Caro P, Richards DE, King KE, Cowling RJ, Murphy GP, Harberd NP (1997). The Arabidopsis *GAI* gene defines a signaling pathway that negatively regulates gibberellin responses. Genes Dev.

[CR32] Richter R, Behringer C, Müller IK, Schwechheimer C (2010). The GATA-type transcription factors GNC and GNL/CGA1 repress gibberellin signaling downstream from DELLA proteins and PHYTOCHROME-INTERACTING FACTORS. Genes Dev.

[CR33] Salanenka Y, Verstraeten I, Löfke C, Tabata K, Naramoto S, Glanc M, Friml J (2018). Gibberellin DELLA signaling targets the retromer complex to redirect protein trafficking to the plasma membrane. Proc Natl Acad Sci USA.

[CR34] Schindelin J, Arganda-Carreras I, Frise E, Kaynig V, Longair M, Pietzsch T, Preibisch S, Rueden C, Saalfeld S, Schmid B, Tinevez JY, White DJ, Hartenstein V, Eliceiri K, Tomancak P, Cardona A (2012). Fiji: an open-source platform for biological-image analysis. Nat Methods.

[CR35] Seaman MN, Marcusson EG, Cereghino JL, Emr SD (1997). Endosome to Golgi retrieval of the vacuolar protein sorting receptor, Vps10p, requires the function of the VPS29, VPS30, and VPS35 gene products. J Cell Biol.

[CR36] Shimada T, Koumoto Y, Li L, Yamazaki M, Kondo M, Nishimura M, Hara-Nishimura I (2006). AtVPS29, a putative component of a retromer complex, is required for the efficient sorting of seed storage proteins. Plant Cell Physiol.

[CR37] Strader LC, Ritchie S, Soule JD, McGinnis KM, Steber CM (2004). Recessive-interfering mutations in the gibberellin signaling gene *SLEEPY1* are rescued by overexpression of its homologue, *SNEEZY*. Proc Natl Acad Sci USA.

[CR38] Thazar-Poulot N, Miquel M, Fobis-Loisy I, Gaude T (2015). Peroxisome extensions deliver the Arabidopsis SDP1 lipase to oil bodies. Proc Natl Acad Sci USA.

[CR39] Tu Y, Seaman MNJ (2021). Navigating the controversies of retromer-mediated endosomal protein sorting. Front Cell Dev Biol.

[CR40] Ubeda-Tomás S, Swarup R, Coates J, Swarup K, Laplaze L, Beemster GT, Hedden P, Bhalerao R, Bennett MJ (2008). Root growth in Arabidopsis requires gibberellin/DELLA signalling in the endodermis. Nat Cell Biol.

[CR41] Ubeda-Tomás S, Federici F, Casimiro I, Beemster GTS, Bhalerao R, Swarup R, Doerner P, Haseloff J, Bennett MJ (2009). Gibberellin signaling in the endodermis controls Arabidopsis root meristem size. Curr Biol.

[CR42] Ueguchi-Tanaka M, Nakajima M, Katoh E, Ohmiya H, Asano K, Saji S, Hongyu X, Ashikari M, Kitano H, Yamaguchi I, Matsuoka M (2007). Molecular interactions of a soluble gibberellin receptor, GID1, with a rice DELLA protein, SLR1, and gibberellin. Plant Cell.

[CR43] Willige BC, Ghosh S, Nill C, Zourelidou M, Dohmann EM, Maier A, Schwechheimer C (2007). The DELLA domain of GA INSENSITIVE mediates the interaction with the GA INSENSITIVE DWARF1A gibberellin receptor of Arabidopsis. Plant Cell.

[CR44] Zelazny E, Santambrogio M, Pourcher M, Chambrier P, Berne-Dedieu A, Fobis-Loisy I, Miege C, Jaillais Y, Gaude T (2013). Mechanisms governing the endosomal membrane recruitment of the core retromer in Arabidopsis. J Biol Chem.

[CR45] Zentella R, Zhang ZL, Park M, Thomas SG, Endo A, Murase K, Fleet CM, Jikumaru Y, Nambara E, Kamiya Y, Sun TP (2007). Global analysis of della direct targets in early gibberellin signaling in Arabidopsis. Plant Cell.

[CR46] Zhu Y, Zhao Q, Cao W, Huang S, Ji C, Zhang W, Trujillo M, Shen J, Jiang L (2023) The plant-unique protein DRIF1 coordinates with SNX1 to regulate membrane protein homeostasis. Plant Cell:koad227 10.1093/plcell/koad227 PMC10689196 37647529

[CR47] Zia SF, Berkowitz O, Bedon F, Whelan J, Franks AE, Plummer KM (2019). Direct comparison of Arabidopsis gene expression reveals different responses to melatonin versus auxin. BMC Plant Biol.

